# Population, Behavioural and Physiological Responses of an Urban Population of Black Swans to an Intense Annual Noise Event

**DOI:** 10.1371/journal.pone.0045014

**Published:** 2012-09-14

**Authors:** Catherine J. Payne, Tim S. Jessop, Patrick-Jean Guay, Michele Johnstone, Megan Feore, Raoul A. Mulder

**Affiliations:** 1 Department of Zoology, University of Melbourne, Victoria, Australia; 2 Wildlife Conservation and Science, Zoos Victoria, Victoria, Australia; 3 Institute for Sustainability and Innovation, Victoria University, Victoria, Australia; University of Lethbridge, Canada

## Abstract

Wild animals in urban environments are exposed to a broad range of human activities that have the potential to disturb their life history and behaviour. Wildlife responses to disturbance can range from emigration to modified behaviour, or elevated stress, but these responses are rarely evaluated in concert. We simultaneously examined population, behavioural and hormonal responses of an urban population of black swans *Cygnus atratus* before, during and after an annual disturbance event involving large crowds and intense noise, the Australian Formula One Grand Prix. Black swan population numbers were lowest one week before the event and rose gradually over the course of the study, peaking after the event, suggesting that the disturbance does not trigger mass emigration. We also found no difference in the proportion of time spent on key behaviours such as locomotion, foraging, resting or self-maintenance over the course of the study. However, basal and capture stress-induced corticosterone levels showed significant variation, consistent with a modest physiological response. Basal plasma corticosterone levels were highest before the event and decreased over the course of the study. Capture-induced stress levels peaked during the Grand Prix and then also declined over the remainder of the study. Our results suggest that even intensely noisy and apparently disruptive events may have relatively low measurable short-term impact on population numbers, behaviour or physiology in urban populations with apparently high tolerance to anthropogenic disturbance. Nevertheless, the potential long-term impact of such disturbance on reproductive success, individual fitness and population health will need to be carefully evaluated.

## Introduction

Wild animals in cities may experience both benefits and costs from living in close proximity to humans. Anthropogenic food sources, warmer microclimates and altered predator-prey dynamics can provide benefits to wildlife [1], [2]. However, animals in urban habitats are also frequently exposed to human-related disturbances, ranging from human presence and approach [3], [4], to various forms of noise [5]–[7] generated by aircraft [8], [9] or other traffic [10], [11]. Understanding what effects such disturbance may have on animals is essential for successful management of urban wildlife and mitigation of any impacts.

Disturbance can have effects at demographic, behavioural and physiological levels. Physical avoidance of a disturbance has been reported in taxa ranging from mountain gazelles *Gazella gazella* [12] to golden plovers *Pluvialis apricaria* [13], pink-footed geese *Anser brachyrhynchus* [14] and moose *Alces alces* [15]. Such avoidance may lead to temporary or permanent reductions in local population size, though few studies have measured short-term population responses to disturbance. Behavioural changes in response to human disturbance include increased vigilance [16], reduced foraging [17], altered parental care [18] and increased movement due to avoidance behaviours [17], [19].

More recently, physiological effects of human activities have also been demonstrated. Several studies have shown changes in heart rate and body temperature, which can occur in the absence of any obvious behavioural responses [20], [21]. In response to disturbance, animals also typically mount a stress response, involving the release of glucocorticoid steroid hormones [22]. The release of stress hormones acts to increase energy available for escape, reduce non-essential activities [7], [23] and modify behaviour to trigger an emergency life-history stage [24]. Elevated glucocorticoid concentrations in response to human disturbance occur in a variety of species including yellow-eyed penguins *Megadyptes antipodes* [25], spotted hyenas *Crocuta crocuta* [26], European pine martens *Martes martes* [27] and white-crowned sparrows *Zonotrichia leucophrys* [28].

How individual species respond to disturbance depends on a combination of factors including disturbance frequency, duration and intensity [7], [29]. Individual and species-specific attributes also influence responses, including variation in temperament [30], [31]. The costs and benefits of each response option influence reactions to disturbance [32], [33]. Previous exposure to the stressor can diminish the extent to which disturbance elicits a response from wildlife. For instance, repeated exposure to a nonlethal stressor can result in tolerance and habituation [8]. Examples of reduced responses due to tolerance include reduced flush distances and reduced glucocorticoid concentrations and are well-documented in areas frequently visited by humans such as beaches [34], ecotourism areas [35], [36] and cities [3], [4], [6]. Waterbirds, in particular, often become tolerant of repeated human disturbance [37].

While urban animals may be capable of adapting to low-level continuous types of disturbance [29], few studies have examined how they respond to short-term, intense disturbances. Given the range of possible responses to disturbance, it is also surprising that behavioural and physiological responses are rarely evaluated in tandem. Since numerous factors can influence disturbance responses, examining behavioural responses in isolation can be misleading [7], [33]. Ideally, several possible responses should be evaluated concurrently, including measurements of the key response variables before, during and after disturbance [38].

Here, we describe a study in which we adopted this integrative approach to investigate possible effects of intense short-term noise disturbance on an urban waterbird population. We compared demographic, behavioural and physiological responses of black swans (*Cygnus atratus*) to the Australian Formula One Grand Prix (GP), an annual car racing event that takes place over four days on a circuit surrounding an urban lake in Melbourne that houses a large population of wild swans. We investigated an array of possible responses to the event, ranging from changes in population size due to emigration of disturbance-sensitive individuals, to potential changes in behaviour, body mass and physiological stress levels estimated from basal and stress response levels of plasma corticosterone. We evaluated these responses against a null expectation of no difference in these parameters for measurements conducted before, during and after the event.

## Methods

### Study Site and Animals

Black swans are large, sexually monomorphic waterbirds. Our study population was at Albert Park Lake, near the Melbourne central business district (37°50′S 144°58′E). Unlike their northern hemisphere counterparts, black swans do not exhibit seasonal migration, and the lake hosts a permanent and largely sedentary population that ranges from 130–210 birds in a given month. Most (∼60%) of individuals in this population have been fitted with neck collars, permitting individual identification [39].

**Figure 1:**
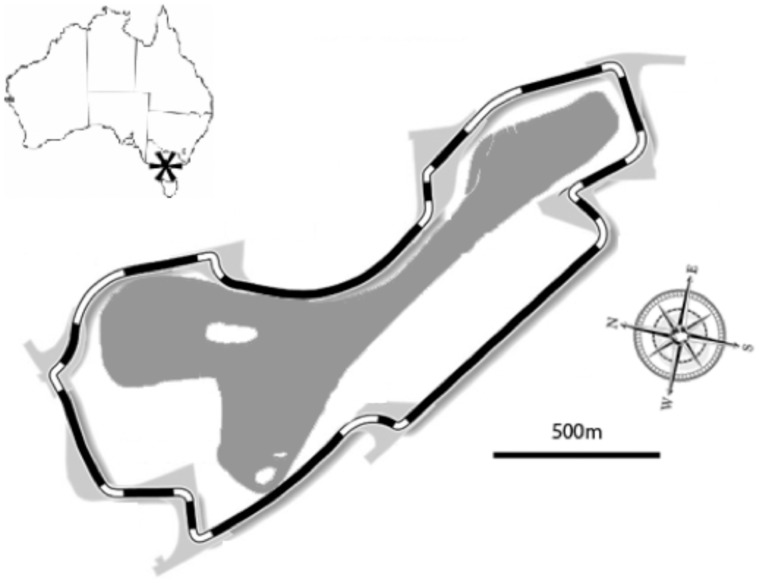
Map of the Grand Prix circuit, illustrating the location of the lake water body (grey) in relation to the racetrack (alternating black and white). Asterisk on the map of Australia depicts the location of Melbourne.

The study population is subject to constant, low-level disturbance due to recreational use of the park and its close proximity to the Melbourne central business district. However, around 300,000 of the estimated five million annual visitors to the park visit during the four days of the GP. The racetrack closely follows the contours of the lake (to within 20 m in some parts; Fig. 1) and the racing vehicles generate noise for up to six hours per day that can exceed 120 dB at 20 m. Gates to the event are opened at 11 am each day and noise intensity is maximal during afternoons, when racing takes place.

We collected data over four separate four-day periods during March and April 2011. The first period was one week prior to the GP (Pre GP; 17–20 March); the second period was during the GP (Grand Prix; 24–27 March); and the last two periods were one (Post GP1; 31 March-3 April) and three weeks (Post GP2; 14–17 April) after the event. We collected data during the morning (0800 h to 1100 h; before spectators entered) and afternoon (1600 h to 1900 h) of each day of each period, to assess whether individuals showed a detectable response for the entire duration of the event, or only during the afternoons, when noise levels peaked.

### Population Counts

We conducted two census counts per day during each sampling period to estimate total population number (eight counts per period). Each census involved circumnavigating the lake and recording all collared and un-collared swans on water and land using a telescope. Morning census counts began at 0800 h and afternoon counts began at 1600 h. Census counts were conducted by one of five different observers, and took around 2.5 hours. We recorded the position of each swan relative to defined landmarks and used these landmarks to section the lake to avoid double counting uncollared birds. All observers used the same landmarks. We began all counts from the same starting point and proceeded anti-clockwise around the lake. The majority of the site is visible from the shore and there is little space for swans to be concealed. This, along with the large size of the swans, made it easy to detect the birds and we assumed a >90% detection rate.

As part of a ‘citizen science’ program [40], a large network of community volunteers (n = 300) monitored waterbodies elsewhere in the Melbourne region for collared swans and submitted records of sightings via an interactive website (myswan.org.au), so that we could confirm any cases of emigration and determine which locations, if any, might be preferred by birds that left the lake. We previously [40] determined that such public reports were highly accurate and reliable.

### Noise Levels

During each census count, we took sound level readings at seven locations spaced evenly around the lake perimeter. Readings were taken for 30 s using a Lutron SL-4001 Sound Level Meter and a slow response measurement with “A” weighting.

### Behavioural Observations

We used focal sampling techniques [41] to conduct 10-minute behavioural observations on individual birds. We selected focal individuals by dividing the perimeter of the lake into numbered sections and selecting a random starting point. We used a coin-flip to determine the direction of our circumnavigation of the lake (clockwise or anti-clockwise) and the sex of the first focal individual. We only observed birds of known sex. We then started walking around the lake from the random starting point in the direction selected, and observed the first bird of that sex visible from the shore. We then moved around the lake observing the next visible individual. We alternated the sex of the focal individual for each observation.

Collared and un-collared swans do not differ in their behaviour [39], so to minimise the probability of repeat sampling of the same individual within an observation period we only observed collared individuals. During each 10-minute observation we scored the focal individual’s behaviour at 10 s intervals. We recorded thirteen types of behaviour, which were subsequently aggregated into five general categories for analysis (Table 1).

**Table 1:** Behaviours recorded for black swan observations at Albert Park Lake.

General categories of behaviour	Detailed behaviours
**Locomotion**	Swimming
	Flying
	Walking
**Foraging**	Dipping with head and neck submerged
	Feeding on water surface (dabbling)
	Upending
	Grazing on land/water’s edge
	Begging for bread
	Drinking
**Resting**	Standing/sitting on land
	Floating on water surface
**Self-maintenance**	Preening
	Flapping wings
	Bathing
	Stretching legs/wings
**Social**	Sexual behaviour
	Antagonistic behaviour
	Gathering nesting material

### Stress Hormone Sampling

We hand-captured swans to collect blood samples to measure body mass and to evaluate basal and captures stress-induced changes in plasma corticosterone. We captured 55 swans across the four sampling periods (Pre GP (n = 20), Grand Prix (n = 12), Post GP1 (n = 9) and Post GP2 (n = 14)). We applied a standardized capture restraint protocol [42] to induce an adrenocortical stress response. This involved capturing swans by hand (<30 seconds), then restraining them by trussing their wings and feet. We collected a (basal) blood sample immediately (within 2 min of capture) and a second (stress response) sample 30 minutes after the first. We collected blood samples from swans between 0800 h and 1600 h. Blood samples (∼400 µL whole blood) were drawn from the tarsal vein using a 27 G heparinised needle and 1 ml syringe. We stored the blood samples on ice for up to four hours before they were centrifuged at 5037 g for five minutes and the plasma separated. We then stored the samples in a freezer at −20C until they were assayed. Prior to release, we recorded the body mass of each swan (to the nearest 0.1 kg, using a spring balance) and head-bill length (nearest 0.1 mm, callipers). We then released each bird at its point of capture. After release, we monitored all individuals briefly (∼5 min) to ensure they displayed normal activity. Consistent with evaluating the density and behavioural effects of the GP on black swans, we applied the capture restraint protocol across the four days of each sampling period, to enable matched physiological evaluation.

### Plasma Steroid Analysis

We measured total plasma concentrations of corticosterone using a commercially available ELISA kit (Cayman Chemical, Michigan, United States of America). Preliminary assays determined that 25 µL of plasma was sufficient for assay use. To minimise cross-reaction and interference, we twice extracted all plasma samples in 3 mL of diethyl ether. To measure the efficiency of extraction we added 20 µl of ^3^H-corticosterone (∼2000 CPM) (MP Biomedicals, Solon, Ohio). To estimate steroid extraction efficiency, 50 µl of each extracted sample was placed into a scintillation vial containing 2 ml of scintillation fluid (Ultima Gold). Sample radioactivity was estimated using a Beckman 2100R Liquid Scintillation Counter. We then took the air dried extracted samples and resuspended them in EIA buffer and refrigerated them overnight at 4°C before assaying them. We followed the Cayman Chemical corticosterone EIA assay procedures without modification to measure plasma corticosterone concentrations. During each assay, we ran samples in duplicate alongside a standard curve of eight known concentrations of corticosterone (5000, 2000, 800, 320, 128, 51.2, 20.5, 8.2 pg/ml).

We calculated final steroid concentrations from standard curves and then corrected for individual sample recovery and addition of ^3^H-corticosterone. Average extraction efficiency for corticosterone over the four assays was 83.4±1.07%. For corticosterone assays, we estimated an intra-assay coefficient of variance of 5.6% and an inter-assay coefficient of variance of 16.0%. To validate the use of the corticosterone EIA kit with swan plasma, we established parallelism between the standard curve and serial dilutions of pooled plasma samples.

### Stress Hormone Response Metrics

We defined four performance measures of glucocorticoid stress responsiveness for black swans. Basal corticosterone response was measured at the time of capture, while the T30 capture stress response reflected plasma corticosterone values after 30 minutes of restraint. In addition we also calculated corticosterone stress responsiveness (CSR; T30 plasma corticosterone value divided by basal plasma corticosterone value) and corticosterone stress magnitude (CSM; T30 plasma corticosterone value minus basal plasma corticosterone value). These four metrics represent putative performance dimensions of an animal’s stress response. Basal corticosterone response is an index of allostatic load, and may also signal chronic stress if basal levels are elevated across the study. The T30 capture stress response value infers the stress state of individuals to the capture stress protocol at a single point in time. CSR is a quotient of stress-induced corticosterone activity which may reveal signal interactions between basal and stress induced corticosterone levels, and CSM is the absolute amount of corticosterone synthesised in response to the capture stress, independent of the basal levels at the time of capture. The last two measures are not commonly considered in field endocrine studies but are intuitively important, as experimental hormone studies in biology and medicine indicate that both the rate and amount (and interaction) of hormone delivery cause differences in hormone-mediated effects on an organism [43]. Both CSR and CSM metrics exclude absolute values at each time period, thus eliminating variance due to analytical variation owing to differences in antibodies, reagents, assays or laboratories.

### Data Analysis

For population counts and behavioural analyses we used Generalized Linear Mixed Models (GLMM) with Poisson error distribution incorporating random effects for observer and repeated observation on known individuals (behavioural analyses only). For hormone data we used GLMM with a log link and Gaussian error distribution, again incorporating random effects for repeated blood sampling of some individual birds across the study. For body mass data we also used GLMM with a log link and Gaussian error distribution, with head-bill length considered as a covariate in this analysis to reduce allometric body mass differences among birds and again incorporating individual identity as a random effect to take into account repeated captures of individuals across the study. We conducted all analyses in IBM SPSS 20. We considered experimental period as a fixed factor in all analyses. We conducted post-hoc pairwise comparisons using Fisher’s protected Least Significant Difference (LSD) tests.

For counts and observations, we also considered time of day (morning vs. afternoon) as an additional fixed factor. We were unable to test for interactive effects of sex and experimental period on corticosterone metrics because during one time period (Post GP1) few females (n = 2) were captured. This unbalanced sample means that we could not accurately estimate error for females for this period. All values reported are mean ± SEM.

## Results

### Noise Levels

Mean noise levels during the GP sampling period (73.0±1.5 dB) were 12–16 dB higher than those measured before (61.0±2.3 dB) or after (56.9±2.2 dB) the event (ANOVA, F_2,20_ = 6.64, p = 0.006), and maximum noise levels recorded during the GP (121.8 dB) were almost twice as high as those recorded before (66.6 dB) or after (73.3 dB) after the event.

### Population Counts

**Figure 2:**
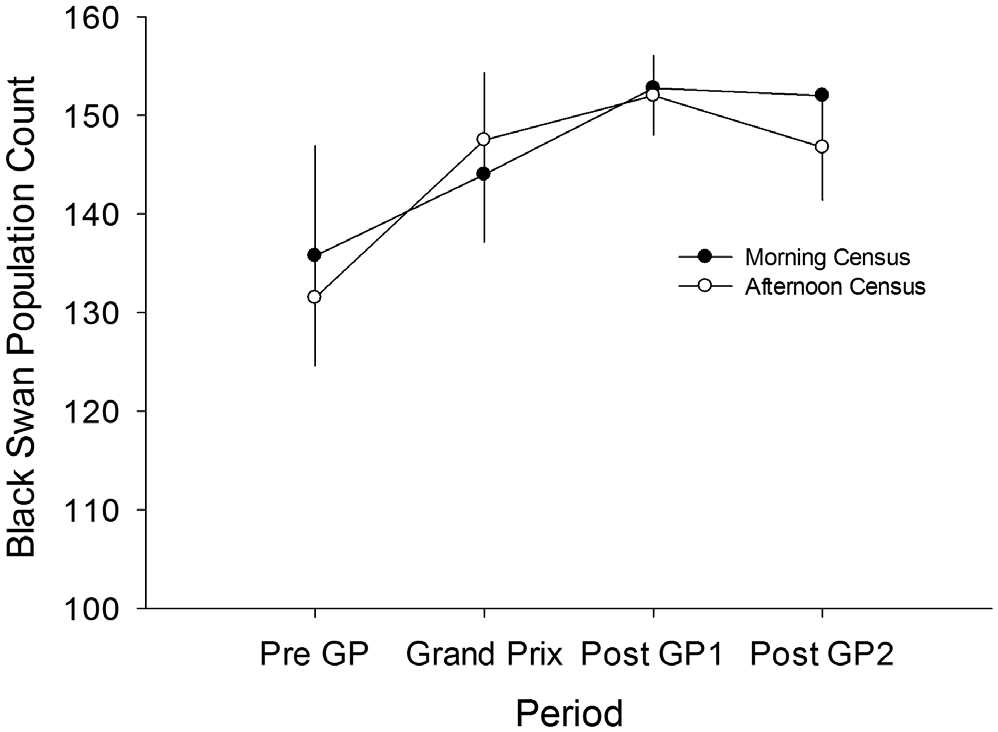
Population counts (mean±SEM) of black swans at Albert Park over the experimental period (n = 8 for all periods). Closed circles represent morning counts and open circles represent afternoon counts.

We used population counts to measure the demographic response of black swans to disturbance effects of the GP over time. Population numbers increased over the course of the study (GLMM, Wald χ^2^_3_ = 9.978, P = 0.019; Fig. 2) from 134±6 individuals one week before the GP to 152±3 individuals one week after the GP. We found no difference between counts conducted during the morning or afternoon (GLMM, Wald χ^2^_1_ = 0.151, P = 0.698; Fig. 2), and there was no significant interaction between experimental period and the time the count was conducted (GLMM, Wald χ^2^_3_ = 1.582, 0.663).

Our citizen scientist network reported 16 sightings of collared black swans elsewhere in the six weeks preceding the GP. Five of these individuals were long-term absentees, for which we received reports from the same location both previously and subsequently. The remaining 11 individuals were long-term residents from the Albert Park population, which briefly emigrated from the site during February and early March. However, all of these individuals returned to the lake before the commencement of the GP and remained there for the duration of the event. Only two individuals were seen elsewhere during the GP. Both individuals were long-term absentees who had been at these sites for some months before the GP and remained there for several months after the event.

### Behavioural Observations

**Figure 3:**
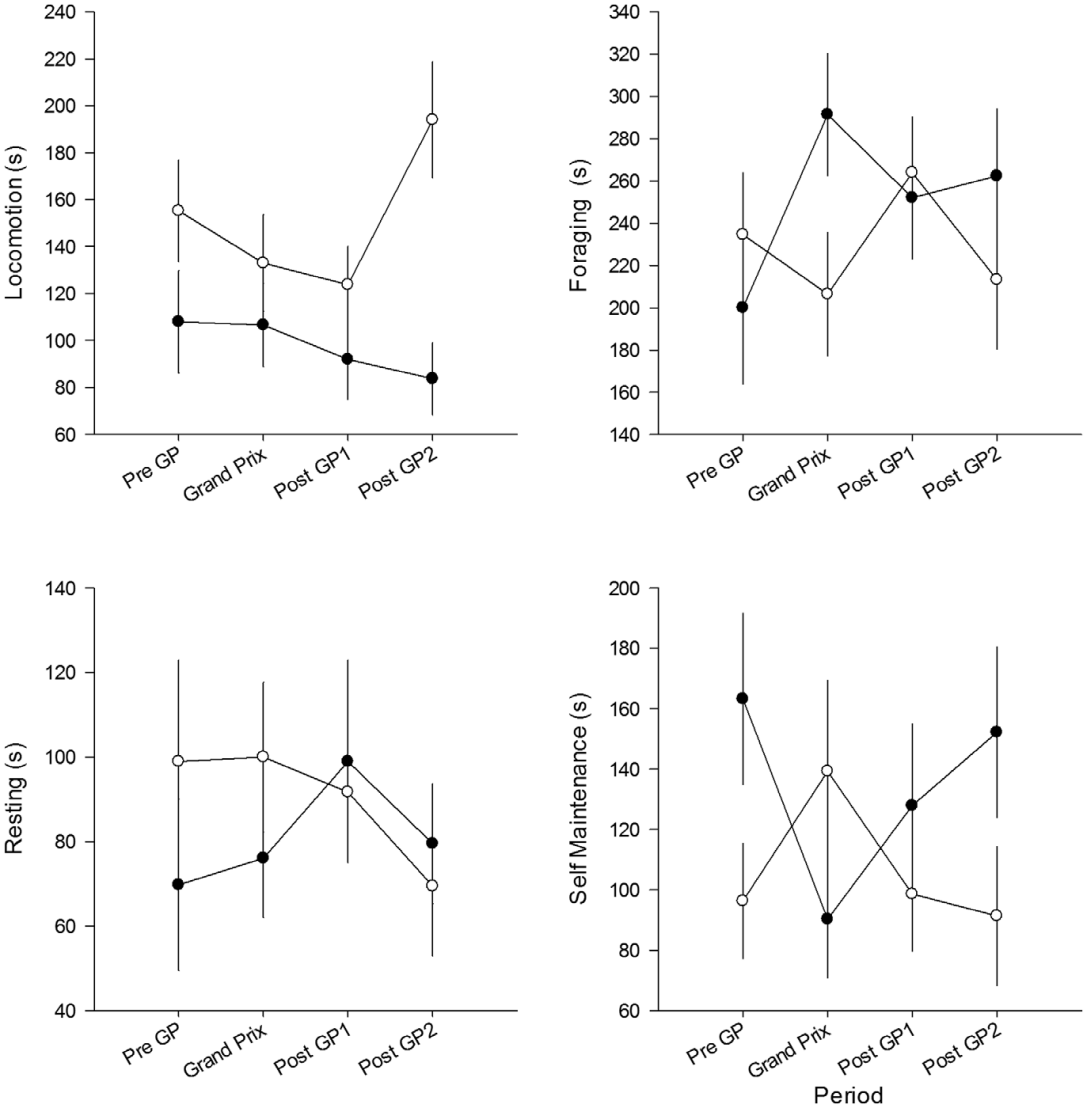
Total time (mean±SEM) spent by black swans on different behaviours during a 10 min observation. Different behaviours observed were a) locomotion, b) foraging, c) resting and d) self-maintenance. Filled circles represent morning observations and open circles represent afternoon observations. Pre GP (n = 83), Grand Prix (n = 82), Post GP1 (n = 89) and Post GP2 (n = 79).

We obtained 333 behavioural observations from 104 individuals, which included over 95% of the collared population present over the course of the study. Only 6 of 333 observations (<2%) involved birds that had been captured the same day, so that there was minimal potential for any effects of capture stress to have influenced behavioural profiles. Overall, swans spent >95% of their diurnal time budget performing four behaviours (foraging, locomotion, resting and self-maintenance; social interactions rarely occurred). We used these four common behaviours as behavioural measures to evaluate the disturbance effects of the GP across time. Over the experimental period, there was no difference in the proportion of time spent on locomotion (GLMM, Wald χ^2^_3_ = 1.829, P = 0.609; Fig. 3a), foraging (GLMM, Wald χ^2^_3_ = 2.333, P = 0.506; Fig. 3b), resting (GLMM, Wald χ^2^_3_ = 1.379, P = 0.711; Fig. 3c) or self-maintenance (GLMM, Wald χ^2^_3_ = 0.439, P = 0.932; Fig. 3d).

When we compared behaviour observed in the morning compared with the afternoon, we found more time was spent in locomotion (GLMM, Wald χ^2^_1_ = 12.583, P<0.001) in the afternoon throughout the experimental period (Fig. 3a). We found no significant differences in time spent on foraging (GLMM, Wald χ^2^_1_ = 0.995, P = 0.318; Fig. 3b), resting (GLMM, Wald χ^2^_1_ = 0.452, P = 0.501; Fig. 3c) or self-maintenance (GLMM, Wald χ^2^_1_ = 2.090, P = 0.148; Fig. 2d) between morning and afternoon. We found no interaction between experimental period and time of observation for amount of time spent on locomotion (GLMM, Wald χ^2^_3_ = 4.301, P = 0.231; Fig. 3a), foraging (GLMM, Wald χ^2^_3_ = 4.728, P = 0.193; Fig. 3b) or resting (GLMM, Wald χ^2^_3_ = 1.868, P = 0.600; Fig. 3c). There was a non-significant interaction between time of day and self-maintenance behaviour (GLMM, Wald χ^2^_3_ = 6.780, P = 0.079; Fig. 3d), with more time spent on self-maintenance in the afternoon compared with the morning of the GP period (GLMM, Wald χ^2^_1_ = 4.596, P = 0.032; Fig. 3d). In the other three periods, more time was spent on self-maintenance in the morning (Fig. 3d).

### Body Mass

There was no indication that swan body mass varied in response to different stages of the GP event (GLMM, Wald χ^2^_3_ = 1.17, P = 0.760).

### Hormone Response

Neither basal nor post-capture corticosterone values were significantly correlated with time of blood sampling (linear regression: basal corticosterone, F_1,53_ = 0.17, P = 0.69; post capture corticosterone F_1,53_ = 0.01, P = 0.97; cubic regression: basal corticosterone, F_2,52_ = 0.55, P = 0.58; post capture corticosterone F_2,52_ = 1.42, P = 0.25).

**Figure 4:**
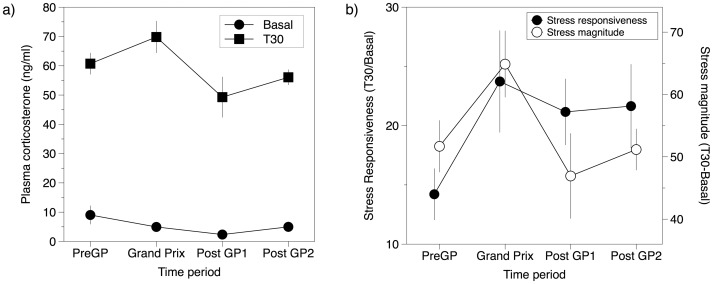
Fluctuations in basal and stress-induced (T30) corticosterone in captured black swans over the period of the study. Panel a) shows plasma corticosterone concentration (ng/ml) of black swans captured before the Grand Prix (Pre GP; n = 20), during the Grand Prix (Grand Prix; n = 12), one week after the Grand Prix (Post GP1; n = 9) and two weeks after the Grand Prix (Post GP2; n = 14) periods. Filled circles represent basal corticosterone levels, filled squares corticosterone levels after 30 min of restraint (T30). Panel b) shows corticosterone stress responsiveness, estimated as T30/Basal corticosterone levels for each time perriod (filled circles) and corticosterone stress magnitude, estimated as T30-Basal corticosterone levels (open circles).

We found a significant effect of experimental period on basal plasma corticosterone, with levels decreasing across time (GLMM, Wald χ^2^_3_ = 9.084, P = 0.028; Fig. 4a). Basal plasma corticosterone levels were highest one week prior to the GP (9.04±3.18 ng/ml) and lowest one week after the GP (2.35±0.22 ng/ml). Post-hoc analysis revealed that corticosterone levels measured one week prior to the GP (Pre GP) were significantly higher (LSD, P = .033) than those in the first week after the GP (Post GP1).

A significant effect of experimental period was also found on T30 post-capture response plasma corticosterone values (GLMM, Wald χ^2^_3_ = 8.164, P = 0.043; Fig. 4a). Capture response plasma corticosterone levels were highest during the GP (69.67±5.44 ng/ml) and lowest at one week post GP (49.33±6.91 ng/ml). There were significant post-hoc pairwise differences between the T30 corticosterone levels measured during the GP and both the first (LSD, P = .020) and second post GP sampling periods (LSD, P = .026).

Corticosterone stress responsiveness, estimated as the rate of increase in plasma corticosterone concentration during capture, exhibited a non-significant trend over time (GLMM, Wald χ^2^_3_ = 7.272, P = 0.064; Fig. 4b). In part this result was due a small increase in corticosterone responsiveness (GLM, Wald χ^2^_1_ = 3.861, P = 0.049; Fig. 4b) in one week prior to the GP (14.51±2.18 ng/mL) followed by a large increase and relatively uniform responsiveness to during the other periods of the study (22–24 ng/ml).

Stress magnitude was calculated as a measure of the level of increase in plasma corticosterone concentration during capture. We found a non-significant trend of stress magnitude over time (GLMM, Wald χ^2^_3_ = 5.987, P = 0.112; Fig. 4b), but a near-significant pairwise difference between the pre-GP (51.70±4.16 ng/mL) and GP stress magnitudes (64.88±5.34 ng/mL) GLM, Wald χ^2^_1_ = 3.791, P = 0.052). Following the GP, the magnitude began to decrease again (Fig. 4b).

## Discussion

Human-generated disturbances are among the most intense selection pressures wild animals must overcome to persist in urban landscapes. Depending on the nature of the disturbance, and prior exposure, wildlife responses may vary from non-responsiveness (implying tolerance), through to partial or large-scale responses including emigration, modified behaviour or elevated stress (implying degrees of intolerance). We did not detect changes in population size consistent with an emigration response to the event, nor changes in behaviour or body mass over the experimental period. However, levels of basal and acute capture stress-induced plasma corticosterone were higher during GP sampling relative to post-GP periods, consistent with disturbance-related increases in physiological stress. Our results suggest that this swan population exhibits behavioural tolerance to this anthropogenic disturbance, but some degree of physiological intolerance, evidenced by elevated stress despite habituation to human presence.

Population counts of swans gradually increased over the duration of the study, peaking one week after the conclusion of the event. Counts were lowest before, rather than during, the event, demonstrating that the event itself does not induce mass emigration of swans from the lake, and fluctuations in population size were within the range recorded during less disturbed times of the year (R. Mulder, unpublished data). Construction-related disturbance associated with the GP commenced several weeks before the event, so it is possible that individuals sensitive to disturbance had already left when we commenced sampling. However, this seems unlikely because annual census counts for February were generally similar to those in March, and our network of citizen scientists did not detect unusually high emigration away from the lake in the six weeks preceding the event. Indeed, all of the swans that temporarily left the lake during the six weeks preceding the event returned to the lake before the GP and remained there during the event. The rise in population size thus probably has a natural underlying cause.

Focal individuals showed unchanged behaviour and body mass over the experimental period. Although the frequency of some behaviours differed with time of day, there was no evidence for systematic interactions between experimental periods and the frequency of particular behaviours. The behavioural profiles observed were similar to those reported in previous studies [39].

In contrast to the lack of behavioural response, basal and capture stress-induced corticosterone levels varied significantly in response to different stages of the GP. Basal corticosterone levels were highest before the GP and subsequently declined. This suggests that it may not have been the event itself (i.e. excessive noise or crowds) that triggered increases in corticosterone levels, but potentially other disturbance stimuli (e.g. construction of infrastructure and road modifications) occurring prior to the race. However, measures of acute capture stress-induced corticosterone levels peaked during the GP. Differing peaks in basal and capture stress-induced corticosterone levels at successive stages indicate different dynamics for each stress metric, and could imply that swans experience periods of both chronic and acute stressor exposure during the staging of the Australian GP.

Peak basal corticosterone levels measured during the Pre GP phase suggest partial attenuation of hypothalamic-pituitary adrenal (HPA) axis, where habituation or downregulation reduces corticosterone synthesis in response to acute stressors [44]. If high basal corticosterone levels and corresponding low levels of corticosterone synthesis in response to capture stress had been maintained throughout the event, this might indicate that the event constituted a chronic physiological stressor to black swans [44]. Instead, we observed a decline in basal titres coupled with either significant or near-significant peaks in the three capture stress-related corticosterone metrics during the GP. This suggests that the event increased acute stress response sensitivity, but that this response was relatively short-lived.

Why did black swans show behavioural, but not physiological tolerance to the event? Animals responding to disturbance must trade off various potential costs to fitness, particularly if responses restrict the opportunity to engage in essential activities such as foraging and breeding [16]–[18], [32]. Flying is energetically expensive [17], particularly for heavy birds such as black swans. Travelling to a less disturbed site can also be costly for individuals if the quality of the new site is unknown and distant [33]. Birds that have invested in activities such as nest building and information acquisition may be less likely to leave despite disturbance [33], especially if the site is perceived to be of high quality.

The lack of any obvious behavioural response of the swans to the GP may have several proximate explanations. Passive low level continuous disturbance is likely to elicit a different response than active high-level intermittent disturbance [29]. The GP event was relatively intense in nature, but short in duration and restricted to the afternoons. The disturbance was also not explicitly targeted at the swans. This may explain why our findings contrast with those of other studies, where the animals themselves are often the target of disturbance and strong responses are typically observed [45], [46]. Proximity to the source of the disturbance may also influence the response of wildlife [38]. Although some parts of the racetrack pass close to the lake’s edge, the unusual geometry of the lake meant that disturbance was mainly restricted to the perimeter of the lake and a small portion in the middle. This may have allowed individuals to avoid the energetic cost of emigrating from the site by instead congregating in ‘refuges’ on the lake where disturbance was minimal [32], [47].

The various costs of responding suggest that animals living in urban environments subject to frequent disturbances should be under selection to develop adaptive responses to disturbance [2], [48]. This commonly manifests as high tolerance to disturbance. Reduced flight distances in response to disturbance have been demonstrated for several urban bird species [3], [4], [6], [49]. Estimates of Flight Initiation distance (FID; the distance at which an animal can be approached before attempting to escape) for black swans vary from 50 to 149 m in low-disturbance areas [30], [50]. The FID of black swans at Albert Park Lake was much shorter at 3.6±3.8 m (mean±SD; [51]) suggesting that individuals in this population are habituated to human disturbance. Habituation has been illustrated in Magellanic penguins *Spheniscus magellanicus*, which showed decreased defensive behaviour after repeated exposure to humans [36]. Conomy *et al.* [8] demonstrated that American black ducks *Anas rubripes* habituated to aircraft noise disturbance, but wood ducks *Aix sponsa* did not, indicating habituation may be species-specific. Urban populations also commonly contain bold and exploratory individuals, which are better able to successfully colonise urban areas [52], [53] and cope with novel situations [6], and this may have contributed to the lack of response observed in our study.

The consequences of brief and sequential periods of chronically and acutely elevated basal and stress induced corticosterone levels during the GP are unknown for black swans. However, it is known that chronic physiological stress can be costly to individuals, as prolonged corticosterone exposure has a myriad of effects on other physiological systems that often negatively affect fitness [7]. In contrast, increased corticosterone levels in response to acute stressors are presumed to be adaptive [23], [24]. It has been argued that animals that are frequently subjected to benign disturbance should mount reduced stress responses in order to minimise the disadvantages associated with chronically elevated glucocorticoid levels [2], [36]. An example of this physiological tolerance is found in some animals exposed to ecotourism. Both Galàpagos marine iguanas *Amblyrhynchus cristatus* and Magellanic penguins in high human visitation areas show a lower stress response to capture and restraint than undisturbed individuals [35], [36]. Lower glucocorticoid concentrations have also been found in urban tree lizards *Urosaurus ornatus* [54] and European blackbirds [2] when compared with rural conspecifics. Partecke *et al.* [2] used a common garden experiment demonstrated that urban blackbirds have a reduced stress response to a standard stressor in comparison to rural conspecifics. A similar finding for behavioural responses to stress has been demonstrated in urban birds, in which variation in flight distance in response to disturbance can be predicted by the number of generations since urbanisation [4]. Nevertheless, the fact that black swans showed both chronic and acute indicators of physiological stress suggests that this species is not completely tolerant to disturbances in their urban environment. Our understanding of what long-term effects, if any, such exposure to stress may have for the individuals concerned is still rudimentary, but recent work has suggested that stressors of similar duration could have potent effects on life history (e.g. survival [55]), especially if exposure occurs during early development [56].

Urban populations are rapidly expanding [57], and urban wildlife continues to be exposed to a range of anthropogenic disturbances. Our results suggest that previous exposure and tolerance of human disturbance may select for attributes in successful urban populations that reduce the short-term impact of novel disturbances, even when these are relatively intense. Specifically, our results imply that black swans are partially habituated to “extreme components” of urban life, given they exhibit no demographic or behavioural responses, and show only modest evidence of physiological stress responses an annual disturbance event. Nevertheless, the potential for longer-term impacts of such disturbance, for instance on reproductive success, individual fitness and population health cannot be ruled out and should be carefully evaluated. For this reason, we advocate integrative studies to identify the full suite of individual and population mechanisms that are possible in response to anthropogenic disturbance.
